# PERSEPHONE: are we ready to de-escalate adjuvant trastuzumab for HER2-positive breast cancer?

**DOI:** 10.1038/s41523-018-0098-y

**Published:** 2019-01-04

**Authors:** Noam Pondé, Richard D. Gelber, Martine Piccart

**Affiliations:** 1 Department of Research, Institut Jules Bordet; Université Libre de Bruxelles, Brussels, Belgium; 2 Department of Biostatistics and Computational Biology, Dana-Farber Cancer Institute, Harvard Medical School, Harvard T.H. Chan School of Public Health, and Frontier Science and Technology Research Foundation, Boston, MA USA

## Adjuvant trastuzumab

**Fig. 1:**
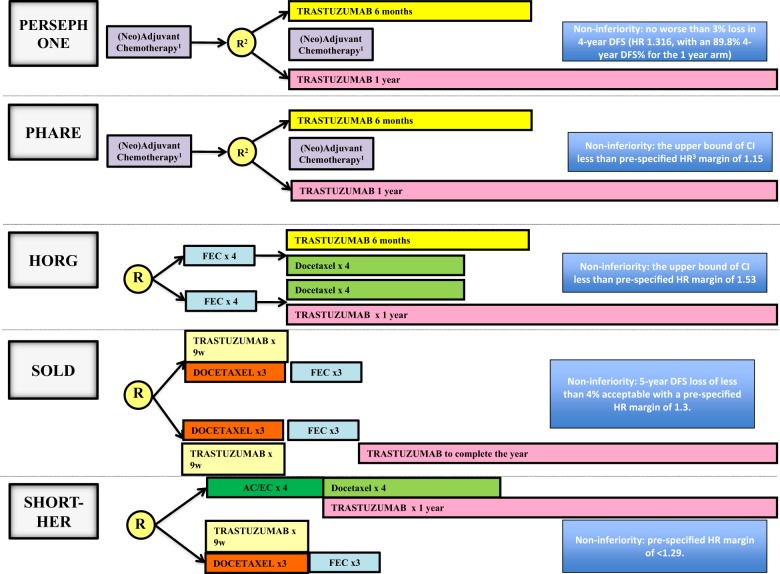
Shorter duration trials—design & definition of non-inferiority. Legend: 1. R randomization, DFS disease-free survival, HR Hazard Ratio, CI confidence interval, FEC 5-fluorouracil, epiribucin, cyclophosphamide, AC anthracyline, cyclophosphamide, EC epirubicin, cyclophosphamide. Trastuzumab could be combined with or sequential with chemotherapy; 2. Patients could be randomised at any point in time up to the 6th month of treatment. 3. The HRs are calculated with shorter arm relative to longer arm, hence the upper bound of confidence interval HR margin greater than 1.00 indicates the maximum increase in relative risk of a DFS event that would be tolerated in order to declare the shorter duration treatment as non-inferior

One year of adjuvant trastuzumab has been the standard treatment for human epidermal growth factor receptor 2-positive (HER2-positive) early breast cancer since the mid-2000s.^1^ From the outset, the expense and cardiac toxicity associated with trastuzumab use led to questioning of treatment duration (1 year), which was chosen based on a slim scientific base. The results of the FinHER trial, which showed tangible benefits with 9 weeks of trastuzumab treatment, suggest that a shorter and cheaper regimen could still be effective.^2^ Later, the lack of added benefit of extended trastuzumab treatment seen in the HERA 2-year arm reinforced the notion of a “ceiling effect”—a point beyond which extending trastuzumab treatment duration does not lead to further improvement in outcomes.^3^ The PHARE,^4^ HORG,^5^ SOLD,^6^ SHORT-HER^7^ and PERSEPHONE^8^ trials were launched to determine whether a shorter regimen would be non-inferior to the standard regimen. Though the first four trial failed to prove non-inferiority, the recently presented non-inferior results of PERSEPHONE have led to considerable debate. Figure 1 depicts the design of these trials and Table 1 summarizes their results.

**Table 1:** Shorter duration trials—results

Trial	Duration of trial^a^	Timing of randomization	Patient characteristics	Chemotherapy with anthracyclines and taxanes	Concomitant trastuzumab with chemotherapy	Patients (*n*)	Efficacy (short arm versus long arm)^b^	Notable Subgroup analysis favouring 1 year	Cardiac events (short arm vs long arm)
*6 months vs 12 months*									
PERSEPHONE^8,15^	8 years	Within first 6 months	N−: 59% ER+: 69%	48%	47%	4089	11.6% vs 11.2% 4-year DFS events HR 1.07 (0.93–1.24)	Taxane-only, concurrent chemotherapy and trend in ER-	9% vs 12%
PHARE^4^	6 years	At 6 months	N−: 55% ER+: 60%	74%	56%	3380	8.9% vs 6.2% 3.5-year DFS events: HR 1.28 (1.05–1.56)	Tumour size >2 cm and sequential chemotherapy-trastuzumab	1.9% vs 5.7%
HORG^5^	8 years	Previously to treatment	N−: 17% ER+: 69%	100%	100%	481	6.7% vs 4.3% 3-year DFS events: HR 1.57 (0.86–2.10)	No significant findings	0 vs 2 cases
*9 weeks vs 12 months*									
SOLD^6^	9 years	Previously to treatment	N−: 60% ER+: 66%	100%	100%	2,176	12.0% vs 9.5% 5-year DFS events: HR 1.39 (1.12–1.72)	lower docetaxel dose, trend in ER- and LN1–3 of benefit in 1-year arm	2.0 vs 3.9%
SHORT-HER^7^	9 years	Previously to treatment	N−: 51% ER+: 67%	100%	100%	1,253	14.6% vs 12.5% 5-year DFS events: HR 1.15 (0.91–1.46)	Stage III and N2/N3 significantly benefit from 1 year	5.1% vs 14.4%

^a^From first patient in to initial presentation of results

^b^The confidence intervals are, respectively, 95% (HORG, PHARE) and 90% (SOLD, SHORT-HER, PERSEPHONE)

## PERSEPHONE

The PERSEPHONE trial, which randomized patients to either 1 year or 6 months of adjuvant trastuzumab, provides the first evidence that a trastuzumab regimen shorter than 1 year could provide non-inferior disease-free survival (DFS). The non-inferiority threshold for DFS was prospectively set as an absolute reduction in 4-year DFS of no more than 3%. Under the investigators’ assumption that the 4-year (4 year) DFS for the 1 year trastuzumab group would be 80%, the 3% decrease in 4-y DFS to 77% corresponded to a hazard ratio (HR) non-inferiority margin of 1.171.

With median follow-up of 5.4 years, 4089 patients and 512 events, the DFS and OS results of PERSEPHONE show non-inferiority between 6 months and 1 year of adjuvant trastuzumab. Four-year DFS was 89.8% in the 1-year arm vs 89.4% in the 6-months arm (HR 1.07, 90% CI 0.93–1.24 *p* = 0.01); four-year OS was 94.8% vs 93.8% (HR 1.14; 90% CI 0.95–1.37).^8^ Cardiac events were also less frequent in the 6-month arm. The subgroup analyses suggest that patients with ER-negative disease, as well as those receiving taxane-based chemotherapy and/or neoadjuvant treatment still need 1 year of trastuzumab.

These intriguing results have led to intense debate on whether 6 months could be considered a new standard, and, additionally, on why PERSEPHONE succeeded when other, very similar trials, failed. Although various small issues can be raised, the central question is the differing definitions of non-inferiority.

## Non-inferiority trials: statistical considerations

Mauri and D’Agostino, Sr. provide a comprehensive and accessible review of the challenges in the design and interpretation of non-inferiority trials.^9^ The presentation in their Table 1 (page 1359) provides explanations on active control, endpoint selection, choice of non-inferiority margin, assay sensitivity, constancy and metrics, execution and analysis, and describes controversies and challenges associated with each of these considerations. For example, in the analysis phase, is it more appropriate to use an intention-to-treat analysis or a per-protocol analysis? If there is treatment crossover or non-adherence, intention-to-treat will tend to bias toward a conclusion of non-inferiority, but a per-protocol analysis may also introduce bias since baseline characteristics are no longer balanced by the randomization process. The choice of one-sided vs two-sided testing procedure, and the appropriate level of statistical significance required to reject the null hypothesis of inferiority are also issues to consider.^10^

The most challenging feature of a non-inferiority trial is prospectively defining a clinically acceptable non-inferiority margin: the amount of reduced effectiveness compared with standard of care that would still be acceptable in light of potential benefit of the new treatment. In most cases, the non-inferiority margin is specified in terms of the relative increase in the risk of the primary efficacy endpoint event associated with use of the new treatment compared with the standard. The question is how much increase in the HR above 1.00 would be acceptable? In PHARE^4^ the investigators prospectively planned non-inferiority would be established if the 6-month treatment were associated with no more than a 15% increase in the relative risk of a DFS event (non-inferiority margin HR of 1.15). To conclude non-inferiority (i.e. to reject the null hypothesis), the upper bound of the two-sided 95% confidence interval resulting from the comparison between the two arms studied in PHARE had to be less than 1.15.

By contrast, the non-inferiority margin for the PERSEPHONE trial was defined in terms of the absolute decrease in the 4-year DFS percent, specifying that the 4-year DFS for the 6-month treatment could be no worse than 3% below the 4-year DFS for the 12-month treatment. Based on data available at the time of study initiation, the investigators estimated that the 4-year DFS for the 12-month duration would be 80%. Using 80% as the control group DFS, the 3% decrease in 4-year DFS (to 77%) corresponded to a 17.1% relative increase in HR, representing a non-inferiority margin of 1.171. Furthermore, the PERSEPHONE investigators specified that a two-sided 90% confidence interval would be used to conclude non-inferiority: this confidence interval is more likely to conclude non-inferiority than the 95% confidence interval specified for PHARE.

In the available results of PERSEPHONE,^8^ the 12-month trastuzumab group 4-year DFS for the population of patients enrolled was 89.8%, considerably better than the 80% estimated at the design stage. As a consequence, the HR non-inferiority margin corresponding to a 3% absolute decrease in DFS percent from 89.8% to 86.8% is 1.316—a 31.6% relative increase in risk of a DFS event. With such a very large margin for acceptable increased risk, it is not surprising that the PERSEPHONE results are reported as demonstrating non-inferiority. The obtained non-inferiority *p*-value of 0.01 indicates that there is a 1% chance of seeing the observed differences in DFS outcomes between treatments, if the true HR comparing 6-months vs 12-months were 1.316 or higher. It is important to highlight that, if the PHARE margin of 1.15 had been used, the PERSEPHONE trial results would not have been statistically significant.

With such a small absolute difference (0.4%) in observed outcomes between the two study arms, it is appealing to suggest that 6 months of trastuzumab might be as good as 12 months. However, this only applies for a population of patients having a risk of a DFS event that is less than 11% by 4 years from enrolment. Thus, the extrapolation of these results to patient populations at higher risk of recurrence, who were not enrolled in PERSEPHONE, remains problematic for the widespread application of the results.

## PERSEPHONE in context

The years since PERSEPHONE was opened saw a number of important changes to the standard of care of early HER2-positive breast cancer, with non-anthracycline regimens (de-escalation) and double blockade/extended therapy (escalation), making PERSEPHONE harder to interpret.^11^

In developing nations, the rationale for the use of the PERSEPHONE regimen is stronger and the use of pertuzumab and neratinib makes little economic sense. It is therefore reasonable to consider the 6 months regimen should be used in most, if not all, situations, in order to spread the benefits of single agent trastuzumab to as many patients as possible.

In developed countries, however, more care should be taken before changing standards. Patients who are at high risk for recurrence and thus candidates for escalated treatment with pertuzumab or neratinib should not receive shortened trastuzumab treatment. Signals coming from the subgroup analysis of the five trials suggest that patients with higher disease burden do derive a higher benefit from the 1-year duration. The same applies to patients who receive neoadjuvant therapy. For patients with low-risk disease—e.g. ER-positive, T1 tumours—de-escalation of chemotherapy should take priority to de-escalation of trastuzumab, and, as the subgroup analysis of PERSEPHONE suggests, for patients receiving taxanes exclusively, 6 months may be inferior to 1 year. Finally, most ER-negative patients are probably not ideal candidates for shorter treatment duration.

Who therefore, should, in developed countries, be treated with 6 months of trastuzumab? Patients with ER+ disease and T2N0 tumours who will not be treated with neoadjuvant therapy are probably the ideal candidates, as long as they receive full anthracycline and taxane regimens. It is important to highlight that more definitive information will be available once an individual-patient level combined analysis is performed, allowing for more robust subgroup analysis.

Seen with 12 years of hindsight, the collective endeavour of the shortened duration trastuzumab trials should be taken as an important lesson. Undoubtedly, the oncology community can ill-afford to design registration trials in the early setting without extensive reflection on the duration of treatment. Designs capable of testing different durations and developing biomarkers capable to differentiate between patients who need no escalation, as well as between those who need different treatment durations should be a standard element of registration trials.^12^ Non-inferiority trials need enormous numbers of patients as well as long-term follow-up and should be run within the context of large international academic consortiums backed by cooperation between governments.^13,14^
